# Diagrammatic dataset on AI-generated formative feedback for XML-based UML models

**DOI:** 10.1016/j.dib.2026.112962

**Published:** 2026-06-13

**Authors:** Janka Pecuchová, Ľubomír Benko, Martin Drlík

**Affiliations:** Faculty of Natural Sciences and Informatics, Constantine the Philosopher University in Nitra, 949 01 Nitra, Slovakia

**Keywords:** Large language models, Automated assessment, Software engineering education, Prompt-based evaluation, UML diagram, AI feedback generation, Multimodal dataset

## Abstract

This dataset describes a diagrammatic, XML-based corpus of student-generated UML models and corresponding AI-generated formative feedback created within a Software Engineering course at Constantine the Philosopher University in Nitra. The released dataset corresponds to one course implementation in the academic year 2024/2025, with data collected and exported in the summer semester of 2025. The release combines 112 anonymized student-level records (student_data25.csv), 448 Slovak-language formative feedback records (feedback_data25.csv) and 700 raw XML reports produced in Enterprise Architect (v16). The XML files encode structural model information such as packages, elements, connectors, stereotypes, attributes, and operations, which were used as the primary machine-readable input for automated evaluation. AI-generated feedback was produced in Slovak language through an OpenAI GPT-4-Turbo (gpt-4-0125-preview) API endpoint available during spring 2025 with temperature 0.3, max_tokens = 700. This linguistic setting provides valuable insights into the performance and adaptability of large language models in non-English educational environments, particularly in technically oriented disciplines. Each record in the dataset links XML-encoded UML models, Slovak-language formative feedback, numeric scores, letter grades, self-reported perceived helpfulness ratings, and corresponding human evaluation. The repository therefore supports research on automated formative assessment, prompt engineering for structured models, human-AI feedback comparison, and multilingual feedback analysis in education. By publishing the dataset structure, prompt templates, evaluation rubric logic, and supporting metadata, the release provides a reproducible basis for future benchmarking, exploratory analysis, and methodological extension in software engineering education.

Specifications Table

**Table:** 

Subject	Computer Sciences
Specific subject area	AI-based formative feedback generation for UML diagram evaluation in software engineering education.
Type of data	Tables (.csv); Structured model files (.xml); Preview images of UML models (.jpg/.png); Markdown/Python source-data files (.md/.py);
Data collection	The public dataset documents one implementation of a university Software Engineering course delivered in the academic year 2024/2025, with all released records originating from the summer semester of 2025. The dataset captures quantitative outcomes, textual feedback, and XML-based UML artifacts related to design-oriented learning tasks. Formative feedback on UML diagrams was conducted through a semi-automated pipeline integrating anonymized data exported from the LMS Moodle with the OpenAI API. The objective was to simulate teacher-like evaluative comments for student UML submissions based on predefined rubrics while maintaining scalability and reproducibility. Following the completion of each assignment, all student submissions were exported using Enterprise Architect (v16, Sparx Systems) as native XML files (.xml), representing the complete internal model structure, including elements, relationships, stereotypes, and attributes. For documentation and human reference, each released XML model was also exported as a corresponding static image file (.jpg/.png), but the AI evaluation process exclusively used the XML representation to access detailed hierarchical and relational data that are not visible in images.
Data source location	Collected: Constantine the Philosopher University in Nitra, Slovakia Stored: Constantine the Philosopher University in Nitra, Slovakia
Data accessibility	Repository name: GitHub Data identification number: 10.5281/zenodo.19037343 Direct URL to data: https://github.com/J-Pecuchova/XML-based-UML-models-dataset The dataset is publicly available on Github.
Related research article	J. Pecuchova, L. Benko, M. Drlik, REIMAGINING FEEDBACK THROUGH GENERATIVE AI IN ENGINEERING EDUCATION, Computers and Education: Artificial Intelligence (2026) 100574. https://doi.org/10.1016/j.caeai.2026.100574.

## 1 Value of the Data

• The dataset provides an authentic, context-bound exploratory benchmark for comparing AI-generated and teacher-generated formative feedback on UML modelling tasks in a university course rather than in a simulated laboratory setting, offering direct comparability between human and AI evaluators without claiming population-level representativeness.
• It combines machine-readable XML exports, preview images of UML models, rubric-based AI feedback texts, numeric scores, letter grades, student-reported perceived helpfulness ratings, and final performance outcome, enabling multimodal analyses across educational, linguistic, and assessment-oriented dimensions.
• The dataset can be used to benchmark XML-based prompt designs for automated feedback generation, compare human and AI feedback styles, or study alignment between rubric-based scores and narrative feedback.
• Because all AI-generated feedback is written in Slovak, the release provides a rare non-English resource for investigating large language model behaviour in low-resource educational contexts. The release is also suitable for reproducibility-focused work because the repository documents the dataset structure, prompt templates, grading rules, and file-level organization needed to rebuild the reported analyses.

## 2 Background

The dataset was compiled to address the challenge of providing scalable, high-quality formative feedback in higher education using Generative Artificial Intelligence (GenAI). Visual modeling tasks, such as designing Unified Modeling Language (UML) diagrams, require expert judgment and detailed commentary, making them difficult to assess efficiently in large classes. The rise and mainstreaming of AI in education in 2023–2025 underscore the urgency of empirical studies comparing human and AI feedback. For example, recent work [1,2] highlights advances in learning analytics for generating personalized feedback at scale and ongoing debates about feedback quality and ethics in automated systems. The growing accessibility of large language models created an opportunity to test their capacity to generate meaningful, formative feedback in complex, open-ended design tasks. The theoretical motivation draws on educational feedback theory [3], dialogic and formative assessment frameworks [4], and cognitive load theory in learning complex representations [5]. Methodologically, the research follows a quasi-experimental mixed-method design combining educational data mining, linguistic analysis, and human–AI comparison to evaluate the quality, sentiment, and pedagogical characteristics of AI-generated feedback. This Data in Brief article complements the related research publication [6]. Its purpose is to document the released files, explain how the data were generated, and improve reuse by clarifying file structure, variable naming, prompt construction, survey scale interpretation, enabling replication, benchmarking, and future research on hybrid human–AI feedback systems.

## 3 Data Description

The public dataset repository (https://github.com/J-Pecuchova/XML-based-UML-models-dataset) provides a structured overview of students’ performance, feedback, and evaluation in a university-level Software Engineering course. The released data correspond to one implementation of the course in the academic year 2024/2025, with all archived submissions, feedback, and evaluation records originating from the summer semester of 2025. As summarized in Table 1, the repository comprises two interlinked CSV files, a collection of raw UML model exports, preview images, and supplementary reproducibility materials. The repository combines data organized at two levels. The CSV files are structured at the participant level and contain 112 unique anonymized student records. In contrast, the XML and image folders are structured at the level of individual UML artifacts. Each student could contribute up to 7 diagram artifacts in total, comprising four semester assignments and three final-exam tasks, which corresponds to a maximum of 784 files (112 students × 7 diagrams). The released repository contains approximately 700 XML model files and 700 corresponding preview images, with a mathing preview image for each released XML artifact. The missing files are due to incomplete submission coverage where some students did not submit all semester assignments, and some did not complete all diagram tasks during the examination. In the exam component, the use case and class diagram artifacts were available for all students, whereas the third artifact was either an activity diagram or a sequence diagram. Most missing exam artifacts are associated with the sequence-diagram task, which many students were unable to solve. In addition, the supplementary folder contains the final prompt templates, a variable-naming and data dictionary, and Python scripts used to reproduce the figures and derived summaries reported in the article.

**Table 1:** Repository structure.

File / Folder	Description	Format	Records / Files	Size (approx.)
student_data25.csv	Quantitative dataset with demographic, performance, and assessment information for all participants.	CSV	112 rows × 37 columns	324 kB
feedback_data25.csv	Textual feedback dataset containing both teacher and AI-generated comments linked to UML assignments.	CSV	112 rows × 45 columns	328 kB
/xml_models/	Raw UML model exports from Enterprise Architect used for AI evaluation.	XML	∼ 700 files	79.1 MB
/images/	Preview visualizations of UML models for reference only, not used as the primary input for AI evaluation.	JPG/PNG	∼ 700 images (one per released XML file)	110 MB
/supplementary_files/	Reproducibility materials including full prompt templates for UML task types, a variable-naming and data dictionary, the Python scripts used to generate the figures and derived summaries reported in the article.	MD/PY	5 files	< 0.1 MB

Researchers can use the dataset in two complementary ways. For exploratory analyses, student_data25.csv can be used to describe cohort characteristics and performance outcomes, while feedback_data25.csv can be joined to it through StudentID to examine feedback type, task-level scores, grades, and perceived helpfulness. For benchmarking work, the XML files should be treated as the primary machine-readable inputs for new automated UML-evaluation pipelines, whereas the corresponding image files provide one-to-one human readable previews for released XML artifacts. The supplementary prompt templates, data dictionary, and scripts can be reused to reproduce the original workflow, compare alternative LLMs or prompt designs with the Slovak-language AI and teacher feedback, and evaluate alignment with human-annotated scores. The dataset should therefore be understood as a context-bound exploratory benchmark rather than as a population-representative benchmark of all software-engineering students or UML modelling tasks.

The dataset student_data25.csv provides the quantitative backbone of the repository (Table 2). Each record represents one anonymized student and summarizes demographic information and performance results obtained through automated quizzes and the final examination. The file serves as a reference for quantitative analyses and can be directly joined with the feedback dataset through the student unique identifier.

**Table 2:** Variables in student_data25.csv.

Variable	Description	Type	Example / Range
StudentID	Unique anonymized student identifier	Integer	290125 – 290386
Y	Study year	Categorical	2 = second-year, 3 = repeating
N	Nationality code	Binary	0 = Slovak, 1 = international
AY	Academic year 2024/2025	Integer	2025
A1–A6	Quiz scores (six theoretical auto-tests)	Integer	0 – 10
A1G–A6G	Corresponding quiz grades	Categorical	‘A’ – ‘Fx’
E	Final exam score	Integer	0 – 10
EG	Final exam grade	Categorical	‘A’ – ‘Fx’

The variables in this dataset (student_data25.csv) enable a detailed exploration of cohort characteristics, study progress, and relationships between feedback exposure and learning outcomes. To provide a quantitative overview of the student cohort’s academic performance, Table 3 summarizes descriptive statistics of all theoretical quizzes (A1–A6) and the final examination (E). For clarity, the values reported in the Max column represent the highest score actually achieved within the observed cohort rather than the maximum possible score. The theoretical quizzes (A1-A6) were graded on a 0–10-point scale, while the final examination (E) was graded on a 0–30-point scale. Consequently, although the maximum observed score in the dataset was 26.33 for the final examination and 9.67 for quiz A5, the full scoring ranges remained 0–30 and 0–10, respectively. These data offer a reference point for interpreting the relationship between AI-generated feedback and subsequent learning outcomes.

**Table 3:** Descriptive statistics of quiz and exam performance.

	Mean	SD	Min	Max
A1	6.70	2.69	0.00	10.00
A2	7.22	2.54	0.00	10.00
A3	7.04	1.52	1.80	10.00
A4	7.52	2.41	0.00	10.00
A5	6.07	3.42	0.00	9.67
A6	2.83	3.73	0.00	10.00
E	16.18	5.13	0.00	26.33

The quiz results demonstrate relatively consistent achievement across theoretical modules (mean (*M)* = 6.23, standard deviation (*SD)* = 1.47), whereas the sixth quiz (A6) displayed the lowest mean and highest variability, suggesting higher task complexity or conceptual difficulty. The final exam (E) exhibited a broader performance range (0–26.33 points), reflecting its integrative design and cumulative assessment structure.

The second file, feedback_data25.csv, contains textual and categorical data describing formative feedback related to student UML modelling tasks (Table 4). To facilitate reuse of the dataset, variable names follow a compact positional naming convention. Prefix S0UX denotes semester UML assignments, whereas E0AX denotes exam-related UML assignments. The placeholder X refers to the specific task or assignment slot within the course workflow. Suffixes encode the variable type, A refers to the assignment variant, F to the generated feedback text, S to the numeric score, G to the letter grade, and LS to the Likert-scale rating of perceived helpfulness.

**Table 4:** Variables in feedback_data25.csv.

Variable	Description	Type	Example / Range
StudentID	Unique anonymized student identifier	Integer	290125 – 290386
G	Feedback condition	Categorical	T / AI
S0UXA	Assignment variant	Categorical	A – F
S0UX	Relative path to submitted UML diagram	String	/models/S0U3/290145_activity.xml
S0UXF	Feedback text	Text	“Add relationship …”
S0UXS	Numeric score for each task	Integer	0 – 10
S0UXG	Corresponding letter grade	Categorical	‘A’ – ‘Fx’
S0UXLS	Likert-scale rating of feedback helpfulness	Numeric	1 – 5
E0AXA	Exam assignment variant	Categorical	A – J
E0AX	UML diagram references for exam submissions	String	/models/E0A2/290145_activity.xml
AM1–AM3	Scores from Annotator M (Teacher 1)	Numeric	0 – 10
MG1–MG3	Grades from Annotator M	Categorical	‘A’ – ‘Fx’
AJ1–AJ3	Scores from Annotator J (Teacher 2)	Numeric	0 – 10
JG1–JG3	Grades from Annotator J	Categorical	‘A’ – ‘Fx’
FG	Final aggregated course grade	Categorical	‘A’ – ‘Fx’

This variable naming scheme is further documented in the data dictionary provided in the public repository. The helpfulness item captures students’ subjective perception of the usefulness of the feedback and should therefore be interpreted as a self-report measure rather than an objective indicator of feedback quality. This file also incorporates final exam results (FG), where submissions were independently evaluated by two annotators (Teacher M and Teacher J), ensuring inter-rater reliability and allowing further studies of assessment consistency.

**Fig. 1:**
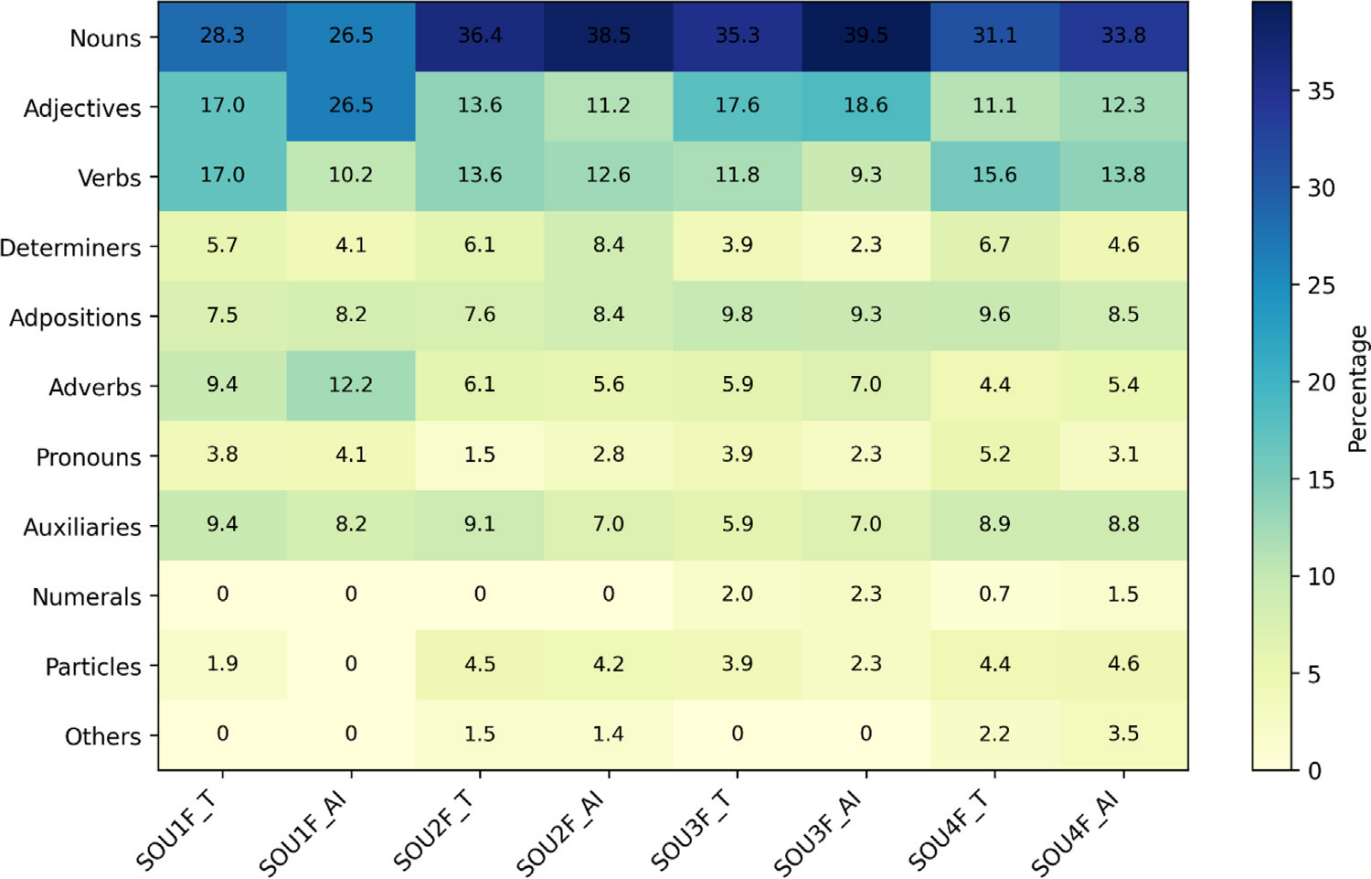
Linguistic characteristics of teacher and GenAI feedback.

Fig. 1 presents the linguistic characteristics of teacher-generated and AI-generated feedback across the four semester UML assignments. Part-of-Speech distributions were computed using the Stanza Python library [7], which supports Slovak morphological annotation. The analysis captures the relative grammatical composition of the feedback texts, including nouns, verbs, adjectives, adpositions, adverbs, auxiliaries and other categories. To complement the POS-based comparison, feedback length was also calculated as word count for each feedback item. Teacher-generated feedback was slightly longer on average, with *M* = 92.72 words, *SD* = 66.18 and *median* = 73.5, while AI-generated feedback reached *M* = 85.06 words, *SD* = 68.75 and *median* = 65.0. This indicates that the POS distribution should be interpreted together with feedback length, since similar grammatical proportions may still reflect different levels of detail or verbosity.

Each record in feedback_data25.csv integrates visual, textual, and evaluative dimensions of the learning process. The dataset is particularly suited for studies examining:

• the linguistic structure and sentiment of teacher vs. AI feedback,
• the relationship between feedback type and subsequent performance, and
• cross-rater grading consistency (quadratic weighted Cohen’s Kappa between human graders higher than 0.90 which represents almost perfect agreement) in design-based assessments.

**Fig. 2:**
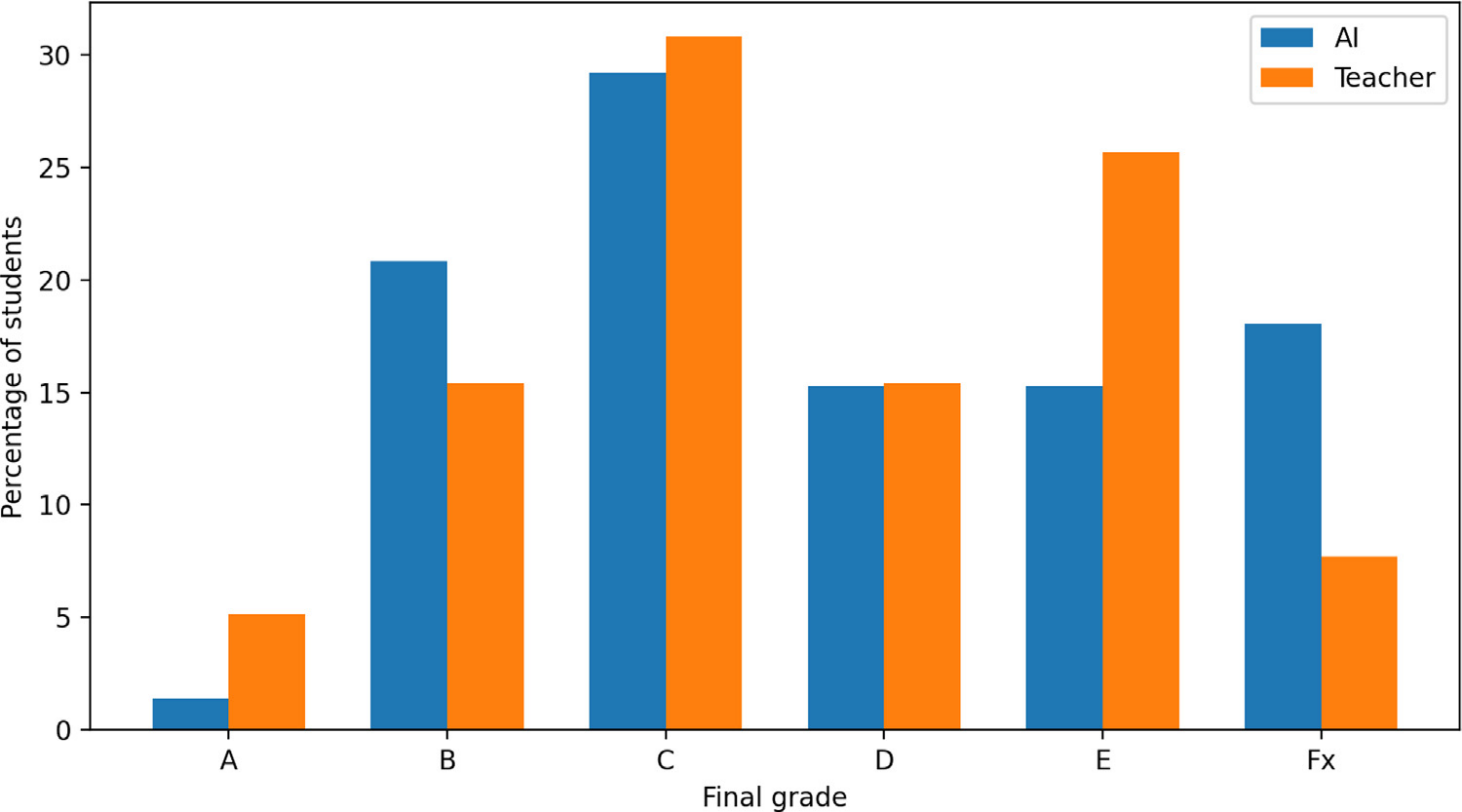
Distribution of final grades by feedback condition.

Fig. 2 presents the distribution of final course grades across the two feedback conditions. The distribution is broadly similar in the middle of the grading scale, with grade ‘C’ representing the most frequent outcome in both groups. The AI-feedback condition shows a higher proportion of ‘B’ grades, approximately 21%, compared with approximately 15% in the teacher-feedback condition. At the same time, the AI-feedback condition also shows a higher proportion of ‘Fx’ grades, approximately 18%, whereas the teacher-feedback condition shows approximately 8%. The teacher-feedback condition contains a higher proportion of ‘A’ and ‘E’ grades, while the proportion of ‘D’ grades is almost identical in both groups. The value of the figure lies in illustrating how the released dataset can be used to relate feedback condition to final grade outcomes and to support further analyses combining performance, feedback text, perceived helpfulness ratings, and task-level UML scores.

## 4 Experimental Design, Materials and Methods

The generation of formative feedback on UML diagrams was conducted through a semi-automated pipeline integrating data exported from the Moodle Learning Management System with the OpenAI GPT-4-Turbo API endpoint (gpt-4-0125-preview). The objective was to simulate teacher-like evaluative comments for student UML submissions based on predefined rubrics while maintaining scalability and reproducibility.

**Fig. 3:**
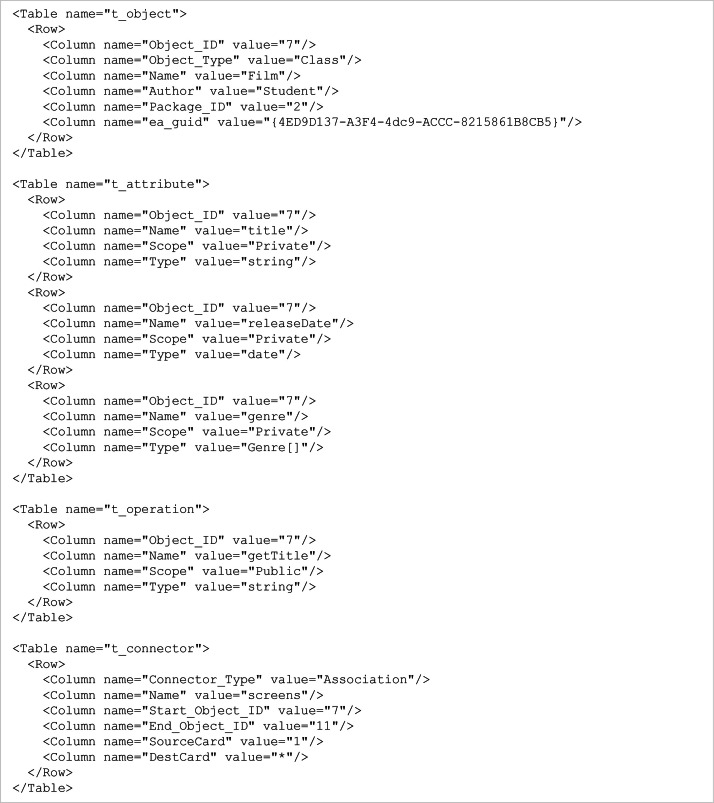
Snippet of XML file produced by Enterprise Architect.

**Fig. 4:**
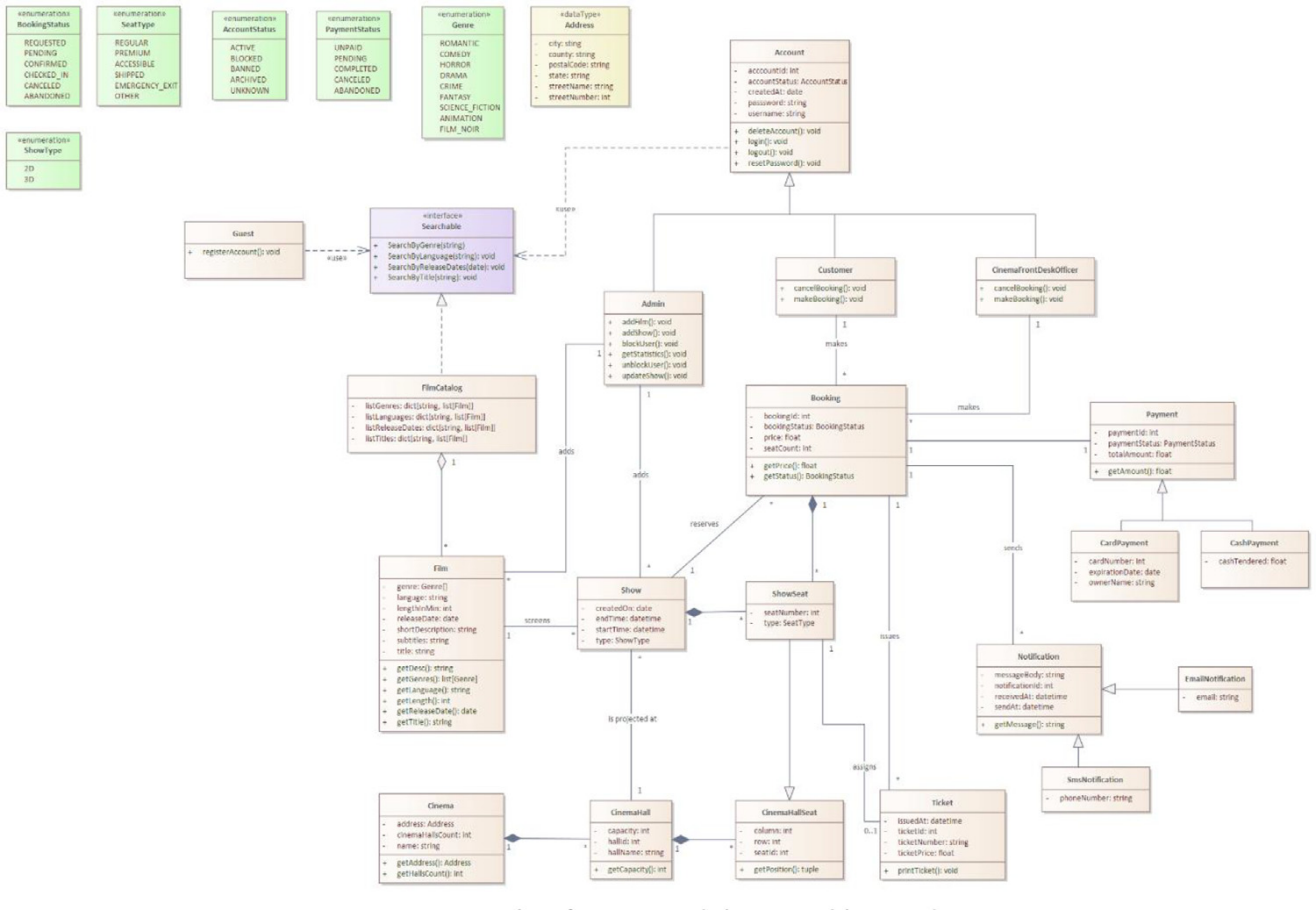
Example of UML model created by students.

Following the completion of each assignment, all student submissions were exported using Enterprise Architect (v16, Sparx Systems) as native XML files (.xml), representing the complete internal model structure, including elements, relationships, stereotypes, and attributes (Fig. 3). For documentation and human reference, each released XML model was also exported as a corresponding static image file (.jpg/.png), but the AI evaluation process exclusively used the XML representation to access detailed hierarchical and relational data that are not visible in images (Fig. 4).

### 4.1 Prompt design and structure

Prompt design was a central part of the feedback-generation workflow because the AI outputs had to be interpretable, rubric-aligned, and sufficiently consistent for subsequent comparison with human evaluation. Each UML task type (Requirements, Use Case, Class, and Activity) was therefore associated with a dedicated prompt template containing four core components: (1) a role definition positioning the model as a university-level UML evaluator, (2) a task-specific evaluation rubric expressed through point-based criteria, (3) explicit output instructions requiring a formative feedback paragraph, a numeric score, and a letter grade, and (4) the XML content of the submitted UML model or its evaluation-relevant excerpt.

The purpose of this structure was not to reproduce human reasoning in a strict cognitive sense, but to standardize the conditions under which the model generated feedback across heterogeneous UML task types. The prompts were designed to direct the model toward structural and semantic properties of the diagrams represented in XML, such as model elements, attributes, operations, connectors, and control-flow constructs, depending on the diagram type. Full final prompt templates for all four UML task types are provided in the supplementary repository materials. The prompt template differed in their grading focus according to the learning objectives of the individual assignments. Requirements models were evaluated with emphasis on the completeness, coherence, and formal quality of requirement definitions. It guided the model to assess whether the XML representation contained a sufficient number of well-structured requirements aligned with the intended system functionality and traceable to higher-level goals.

**Figure:**
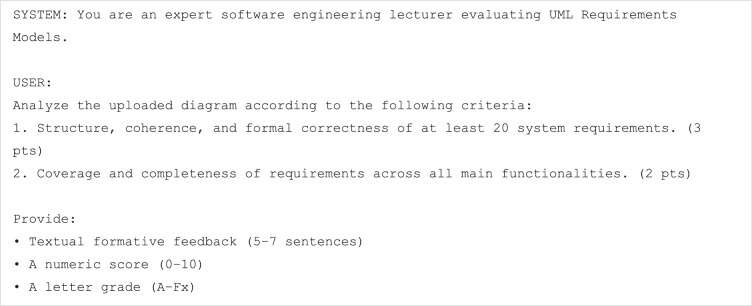
Prompt for requirements model

For Use Case models, the prompt was designed to assess the correctness of actors, use cases, system boundaries, and stereotypical notations («include», «extend»).

**Figure:**
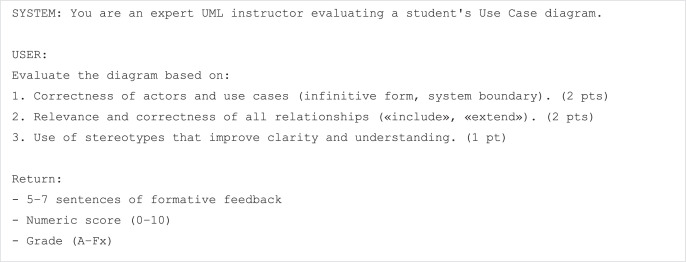
Prompt for use case model

Class diagrams were evaluated primarily through the internal consistency of classes, attributes, operations, and inter-class relationships, including multiplicities where applicable.

**Figure:**
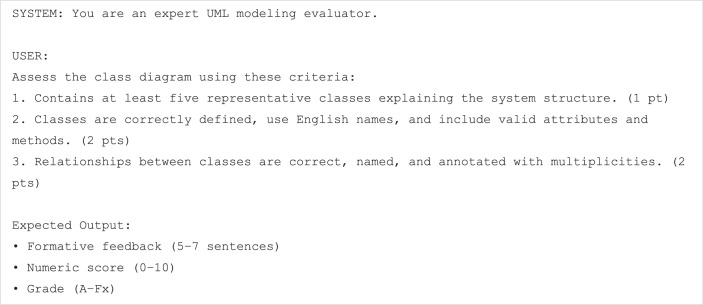
Prompt for class diagram model

Activity Diagrams were assessed in terms of process logic, sequencing of actions, and the correct use of UML activity-model constructs such as initial nodes, final nodes, decisions, merges, forks, and joins.

**Figure:**
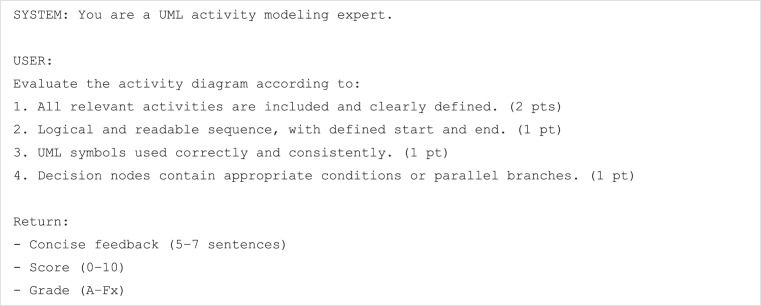
Prompt for activity diagram model

In all cases, the expected output format was standardized where the model was instructed to produce short formative feedback in Slovak (5 – 7 sentences), a numeric score on a 0 – 10 scale, and a corresponding letter grade on the ‘A’ – ‘Fx’ scale. The grading thresholds were defined in accordance with the course rules, whereby grade ‘A’ corresponded to a score between 95.00% and 100.00%, grade ‘B’ to 90.00%–94.00%, grade ‘C’ to 85.00%–89.00%, grade ‘D’ to 80.00%–84.00%, grade ‘E’ to 70.00%–79.00%, and grade ‘Fx’ to 0.00%–69.00%.

### 4.2 Implementation of the evaluation pipeline

The evaluation workflow was implemented in Python 3.10.10 using pandas 2.2.2, xml.etree.ElementTree for XML processing, and the OpenAI Python package (v1.14.2). For each UML submission, the pipeline identified the task type, loaded the corresponding XML file exported from Enterprise Architect, and inserted the XML content into the task-specific prompt template. The workflow operated exclusively on anonymized records and did not transmit personal student identifiers to the API. The feedback generation was performed through the exact GPT-4 Turbo model identifier gpt-4-0125-preview, which was the API endpoint available during the summer semester of 2025 data collection period, this identifier is reported explicitly to support interpretation of future model drift. The generation parameters were set to *temperature* = 0.3 and *max_tokens* = 700 in order to reduce excessive output variability while preserving enough flexibility for natural-language formative comments. The workflow can also be adapted to other generative AI models, subject to their respective context-window and input-format constraints. The system iterated over each XML, constructed the corresponding task-specific evaluation prompts, and submitted the XML representation together with the assessment instructions to the model. Two use-case diagram files 290207_usecase.xml and 290153_usecase.xml, constitute size outliers and cannot be accommodated in their entirety together with the prompt instructions, assessment criteria, and reserved output tokens within the context window of the model used in this pipeline. These files therefore require a separate preprocessing branch. First, the XML is parsed using xml.etree.ElementTree, and a compact structured representation is created by extracting complete UML elements and their semantic relationships, including actors, use cases, associations, stereotypes, and include, extend, and generalization connectors. If the structured representation still exceeds the available input capacity, it is partitioned into logically coherent batches while preserving each element together with its directly connected relationships. The batches are processed independently using the same task-specific prompt and assessment criteria, and their outputs are subsequently consolidated into a single diagram-level evaluation. Fixed-length character truncation is not used for these files because it could divide XML structures or omit semantically relevant UML elements. The complete, untruncated XML files are retained in the released dataset.

**Figure:**
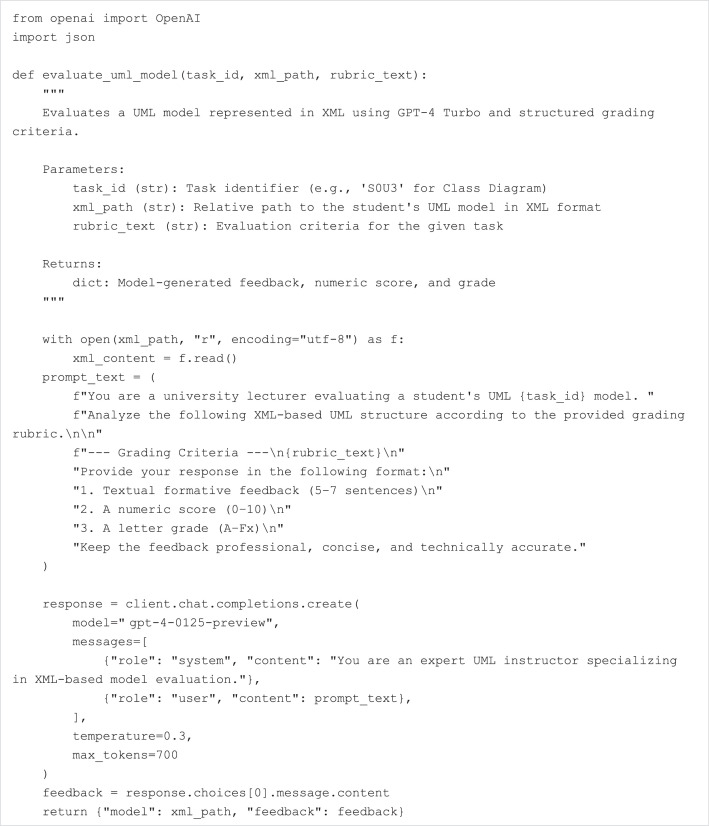
Structured grading prompt for UML diagrams

The following example illustrates a typical model response (translated by authors into English) for a class diagram evaluation:



The generated feedback was linked with student data (student_data25.csv) via anonymized identifiers. After each semester-task, students assigned a single helpfulness rating on a five-point ordinal scale. The exact Slovak item wording was: *„Do akej miery Vám spätná väzba pomohla pochopiť, čo ste urobili správne a čo môžete prípadne zlepšiť?“ (“To what extend did the feedback help you understand what you did correctly and what you could improve?”)* The scale anchors were: 1 = *„Vôbec mi nepomohla“ (“It did not help me at all”)*, 2 = *„Pomohla mi len minimálne“ (“It helped me only minimally”)*, 3 = *„Pomohla mi čiastočne“ (“It helped me partially”)*, 4 = *„Pomohla mi do určitej miery“ (“It helped me to some extent”)*, and 5 = *„Veľmi mi pomohla“ (“It helped me a lot”)*. Within the dataset, this variable represents an ordinal self-report measure of perceived helpfulness and should not be interpreted as an objective indicator of learning improvement.

## Limitations

Although the dataset provides a rich multimodal foundation for analyzing automated feedback generation in software engineering education, several limitations should be acknowledged. First, the released data originate from a single institution and one course context, which limits direct generalizability across different educational settings or disciplines. The release should therefore be understood as a context-bound exploratory benchmark that can support further evaluation and comparison, rather than as a population-representative benchmark. Second, the AI-assisted feedback generation pipeline relied on XML exports from Enterprise Architect as its primary machine-readable input. While this representation allows precise structural parsing, it may not fully capture the visual or stylistic nuances of student modeling practices. Differences in how students structure their models, naming conventions, hierarchy depth, or level of abstraction, could affect AI evaluation consistency. By contrast, human evaluators could inspect the full model directly and interpret it in its broader modeling context. Third, some student submissions contained a mix of Slovak and English terminology, whereas all AI-generated feedback was consistently produced in Slovak. Large language models demonstrate varying degrees of fluency and precision across languages, and subtle differences in translation, terminology, or phrasing could have influenced the accuracy, tone, or granularity of the generated feedback. The possible effect of this linguistic mixture on AI scoring accuracy or feedback relevance was not quantified separately in the present release. No systematic semantic edge-case anomalies attributable to the cross-language instruction mapping were identified during manual checking of the released textual data, although minor translation-related variation in UML terminology or phrasing cannot by fully excluded, particularly in mixed Slovak-English submissions. Finally, the released dataset focuses on instructional artifacts, feedback outputs, and related performance indicators, and it does not provide long-term longitudinal tracking of student learning development beyond the observed course context.

## Ethics Statement

This research involved the analysis of anonymized student submissions and feedback data collected within the framework of higher education coursework at the University of Constantine the Philosopher in Nitra. All data were obtained from institutional Learning Management System (Moodle) and consisted exclusively of anonymized student-generated artifacts (text and image-based UML diagrams) and voluntary self-evaluation responses. No personal identifiers, demographic details, or login information were included in the released dataset. Participation in the learning activities was part of regular coursework, and the subsequent data processing for research purposes was performed only after anonymization at the institutional level. All participants were informed that their submissions could be used for research and educational quality improvement under conditions of full anonymity and non-traceability of individual identities. Given the non-invasive nature of the study and the absence of personally identifiable information, individual written consent was not required. The study protocol, including anonymization procedures and data processing workflow, was reviewed and approved by the Ethics Committee of the University of Constantine the Philosopher in Nitra (approval number UKF/225/2024/191013:002). All procedures adhered to the ethical principles of the Declaration of Helsinki and institutional policies on research involving human participants in educational contexts. The authors confirm compliance with all ethical and publication requirements of *Data in Brief*.

## Credit Author Statement

**Janka Pecuchová:** Conceptualization, Methodology, Software, Data curation, Writing – Original draft preparation, Writing – Reviewing and editing, Supervision, Project administration, Funding acquisition. **Ľubomír Benko:** Conceptualization, Methodology, Software, Investigation, Writing – Original draft preparation, Writing – Reviewing and editing, Visualization, Formal analysis. **Martin Drlík:** Conceptualization, Methodology, Software, Data curation, Writing – Reviewing and editing, Supervision.

## Data Availability

GitHubXML-based-UML-models-dataset (Original data).
